# A Novel Approach for Non-Invasive Continuous In-Line Control of Perfusion Cell Cultivations by Raman Spectroscopy

**DOI:** 10.3389/fbioe.2022.719614

**Published:** 2022-04-25

**Authors:** A. Graf, J. Lemke, M. Schulze, R. Soeldner, K. Rebner, M. Hoehse, J. Matuszczyk

**Affiliations:** ^1^ Product Development, Sartorius Stedim Biotech GmbH, Göttingen, Germany; ^2^ Corporate Research, Sartorius Stedim Biotech GmbH, Göttingen, Germany; ^3^ Process Analysis and Technology PA&T, Reutlingen University, Reutlingen, Germany

**Keywords:** continuous manufacturing, Raman spectroscopy, automated glucose control, CHO perfusion process, quality by design (QbD), process analytical technologies (PAT)

## Abstract

Continuous manufacturing is becoming more important in the biopharmaceutical industry. This processing strategy is favorable, as it is more efficient, flexible, and has the potential to produce higher and more consistent product quality. At the same time, it faces some challenges, especially in cell culture. As a steady state has to be maintained over a prolonged time, it is unavoidable to implement advanced process analytical technologies to control the relevant process parameters in a fast and precise manner. One such analytical technology is Raman spectroscopy, which has proven its advantages for process monitoring and control mostly in (fed-) batch cultivations. In this study, an in-line flow cell for Raman spectroscopy is included in the cell-free harvest stream of a perfusion process. Quantitative models for glucose and lactate were generated based on five cultivations originating from varying bioreactor scales. After successfully validating the glucose model (Root Mean Square Error of Prediction (RMSEP) of ∼0.2 g/L), it was employed for control of an external glucose feed in cultivation with a glucose-free perfusion medium. The generated model was successfully applied to perform process control at 4 g/L and 1.5 g/L glucose over several days, respectively, with variability of ±0.4 g/L. The results demonstrate the high potential of Raman spectroscopy for advanced process monitoring and control of a perfusion process with a bioreactor and scale-independent measurement method.

## Introduction

Within the next years, the biopharmaceutical industry will face several challenges due to changing markets. Several prominent blockbuster drugs have already lost their patent protection, and others will follow soon, while at the same time, it is becoming harder to find new promising candidates (Walsh, 2018). This is forcing industry leaders to adapt to new trends, such as personalized medicine, but also offers great opportunities for biosimilar producers. These changes force the industry to become more flexible towards lower production volumes and reduced turnover times between different production processes, thus causing higher cost pressure (Levine et al., 2012; Klutz et al., 2015; Lu et al., 2017). Here, continuous bioprocessing offers many advantages over traditional (fed-) batch manufacturing.

While fed-batch processes are still the most common practice in biopharmaceutical production, recent studies have shown that continuous manufacturing can significantly improve production efficiency. One particular study calculated that continuous processing potentially reduces the cost of production by about 50% (Yang et al., 2019). One reason for this substantial decrease in cost is the intensification of the process in terms of high viable cell densities or extended culture duration and the resulting significant improvement in productivity (Xu et al., 2020). Simultaneously, the product quality can also be enhanced due to a more stable operation in a steady-state (Karst et al., 2017). Other advantages are the redundancy of the costly and timely scaling of the clinical to the manufacturing scale, as scaling for higher drug product volume can be achieved by either prolonging the manufacturing run or parallel operation of several continuous vessels. This results in a much smaller footprint in terms of equipment, energy, and (raw) materials, as well as facilities needed for production and reduced time to market (Croughan et al., 2015; Klutz et al., 2015; Hernandez, 2017). Finally, regulatory agencies, such as the FDA, are now promoting continuous over batch manufacturing (Langer and Rader, 2014; Lee, 2017).

On the contrary, continuous bioprocessing bears many challenges itself that must be addressed. One aspect is the increased risk of contamination caused by prolonged process times and the resulting increase of samples taken per batch. The longer processing duration also leads to a greater danger of hardware failure. Another issue is cell line instability, in the sense that due to naturally occurring genetic changes within the cell line over the prolonged cultivation period, the productivity, or worse the product quality, may decrease (Croughan et al., 2015; Hernandez, 2017).

These risks can be mitigated by following appropriate process development principles, e.g., quality by design, as well as the thorough implementation of advanced process analytical technologies and process automation. One previously investigated technology is the application of bio-capacitance sensors, such as the BioPAT^®^ Viamass, for automated cell bleeding and therefore maintaining a constant viable cell density within the bioreactor (Grieb et al., 2019). Berry et al. have shown that the product quality can be improved by lowering the glucose concentration to around 2 g/L and maintaining it at a constant level there (Berry et al., 2016). This results in significantly reduced glycation of the antibody. Glycation can, in some cases, inhibit the antibody from binding to the desired antigen, or even worse, cause the antibody not to work at all and possibly causing an immunogenic response (Berry et al., 2016; Beyer et al., 2018). For these reasons, the implementation of an in-line glucose analyzer for monitoring and control is beneficial. Traditionally, glucose is measured manually off-line with blood-gas or similar bio-analyzers. Those typically utilize electro-chemical sensors that for example measure the enzymatic oxidation of glucose (Villena Gonzales et al., 2019). For on-line measurements also electro-chemical sensors are available that can quantify glucose with high accuracy. On the contrary these are susceptible to fouling of the electrodes in prolonged bioprocesses such as perfusion and other continuous cultivation methods (Holzberg et al., 2018). Alternatively, different types of optical sensors can be implemented in-line. Here, spectroscopic methods excel, as they can be integrated non-invasively directly into the process vessel or cell-free perfusion harvest stream. In combination with multivariate methods, an in-line prediction of the current analyte concentration is possible. Advantageously no consumables are necessary, and therefore no maintenance of the sensor is needed even in extended cultivations (Holzberg et al., 2018).

Several different spectroscopic methods suitable for glucose measurement in mammalian cell cultures, i.e., Raman, Infrared, and 2D-Fluorescence, have been identified and compared in different studies (Rowland-Jones et al., 2017; Holzberg et al., 2018). As glucose does not fluoresce, 2D-Fluorescence can detect this parameter only by correlation to other analytes, which makes it highly vulnerable to process deviations. Near-Infrared (NIR) and Raman spectrometry detect glucose directly and are therefore better suited. Between the two, Raman has shown to have a better model performance, resulting in lower prediction errors than NIR (Rowland-Jones et al., 2017). Mid-Infrared (MIR) spectroscopy can detect glucose with similar sensitivity as Raman yet lacks commercial availability when it comes to in-line measurements especially in single-use operations (Graf et al., 2021).

Raman spectroscopy is based on inelastic scattering of incident light on certain molecules. This Raman shift is highly specific to the molecules’ structures, creating a molecular fingerprint of the sample. Furthermore, since the effect is proportional to the concentration of the molecules, quantification of certain analytes of interest is possible (Bakeev, 2010). However, Raman scattering occurs extremely seldom, therefore a relatively strong laser is necessary to increase the chance of single photons to undergo inelastic scattering. In case of bioprocessing the wavelength of the excitation laser is usually in the near-infrared region to minimize unwanted disturbance of fluorescence (Esmonde-White et al., 2017). Nevertheless, the useful Raman signal is comparably weak to the fluorescence background which also changes during the course of a cultivation due to accumulation of fluorophores through feed media addition and cell metabolism. Additionally, other negative influences, such as interference by high cell counts, can make the interpretation of a spectrum challenging. To counteract these effects, data pre-treatment is usually necessary. Depending on the process and utilized equipment, this can involve methods such as baseline correction, derivative transformation, normalization, noise removal, and smoothing algorithms (Buckley and Ryder, 2017). In combination with multivariate methods, such as Principal Component Analysis (PCA) and Partial Least Squares (PLS), the Raman spectra can either be used for qualitative monitoring of a process in the first case, or quantification of nutrients and metabolites in the latter one (De Gelder et al., 2007; Buckley and Ryder, 2017; Esmonde-White et al., 2017).

Even though several in- or on-line solutions, such as the ones discussed above, are available today, a majority of commercial bioprocesses still rely on manual daily sampling. With the exception of physical parameters, such as pH, temperature and dissolved oxygen, that are already controlled automatically, most biological parameters like cell count, viability, nutrient, and metabolite concentrations are only measured at-line once or twice a day. This makes process control difficult and increases the risk of batch failure (Gillespie et al., 2022; Reyes et al., 2022). Various studies have investigated how Raman spectroscopy can be used as a PAT tool for on-line measurement of glucose and how a control loop for this parameter can be established. Craven et al. developed a non-linear model predictive controller, that in combination with on-line glucose quantification by Raman spectroscopy was able to maintain the process at a fixed glucose concentration (Craven et al., 2014). Hirsch et al. utilized Raman technology for control of an ethanol producing yeast fermentation. The process was kept at around 100 g/L glucose resulting in a significantly higher ethanol yield (Hirsch et al., 2019). The previously mentioned study of Berry et al. also utilized Raman spectroscopy for in-line quantification of glucose. This enabled the authors to establish a feed-back control loop that maintained the glucose concentration within the process around 2 g/L. While good control performance could be achieved during the exponential growth phase of the process, prediction errors increased significantly after attaining peak viable cell density (VCD) (Berry et al., 2016). In a later follow-up study Matthews et al. investigated how Raman spectroscopy with a higher laser wavelength can reduce unwanted interference from process autofluorescence. Here, also a glucose control loop was implemented that maintained the process around 3 ± 0.5 g/L (Matthews et al., 2018).

For all the aforementioned reasons, the authors have chosen to integrate a Raman spectrometer into the harvest line of a perfusion process. This location is favorable because the cell-free perfusion permeate enables optimal capturing conditions without the strong scattering occurring at high cell densities. Additionally, it allows for an analyte measurement independent of utilized bioreactor type as well as scale. Moreover, it makes integration into existing systems more straightforward, especially in single-use applications, as no redesign of the bioreactor or cultivation bag is necessary. In this study, five different cultivations were run to build robust analyte models, with a sixth run as an outside dataset to evaluate the models’ performance. In a final perfusion cultivation, the glucose model was used for in-line prediction of the analytes’ concentration. The predicted glucose concentration was used for in-line glucose feedback control, successfully maintaining a constant glucose level within the bioreactor at 4 g/L and 1.5 g/L, respectively.

## Material and Methods

### Cultivations

Perfusion cultivations were performed using an industrial relevant CHO cell line (DG44, Sartorius, Germany) expressing a monoclonal antibody (IgG1). All used media were chemically defined (Sartorius, Germany). Seed medium (SMD) was used for all seed cultures. A previously designed blend of fed-batch media was used as a perfusion medium (PF-M) (Janoschek et al., 2019).

Seed cultures were grown in shake flasks in an incubation shaker at 36.8°C, 7.5% pCO_2_, 85% humidity, and 120 rpm (CERTOMAT^®^ CT plus, Sartorius, Germany) for four passages. Perfusion cultivations were performed using 2 L perfusion bioreactor bags (Flexsafe® RM Perfusion, Sartorius, Germany) with a working volume of 1 L (C2, C5, and C6) or 20 L bags with 10 L working volume (C1, C3, and C4) with a 2-dimensional rocking motion bioreactor (BIOSTAT® RM 20|50, Sartorius, Germany). The bioreactors were inoculated at 0.2 × 10^6^ cells/mL in SMD in batch mode (T = 36.8°C, pH = 7.1, DO = 60%, rocking rate = 30 rpm with 10° angle). The pH-level was controlled by CO_2_ gassing. When a VCD of 2.5 × 10^6^ cells/ml was reached, perfusion was started with a perfusion rate of one vessel volume exchange of medium per day (VVD). Simultaneously, pH was shifted to 6.95, and control was extended by adding 1 M Na_2_CO_3_. Different control strategies for perfusion rate were utilized as described in Table 1. In summary, perfusion rate was either adjusted daily based on expected cell growth for the following day (step-wise increase) or continuously adjusted based on on-line biomass measurement to maintain a constant cell-specific perfusion rate (CSPR). For all cultivations, a minimum perfusion rate of 1 VVD was used.

**TABLE 1:** Overview of perfusion cultivations.

#	Bag scale	Cell bleed level	Perfusion rate	Chemometrics
C1	20 L	–	Adjusted daily max. 3 VVD	Analyte model building
C2	2 L	–	Adjusted daily max. 4 VVD	Analyte model building
C3	20 L	–	Constant CSPR	Analyte model building
			Day 0–5: 50 pL/(cell·d)	
			Day 5–7: 20 pL/(cell·d)	
C4	20 L	–	Adjusted daily max. 3 VVD	Analyte model building
C5	2 L	20–30 × 10^6^ cells/ml	Day 0–8: 1 VVD	Analyte model building
			Day 8–18: 1.25 VVD	
C6	2 L	–	Constant CSPR	Analyte model validation
			50 pL/(cell·d)	
C7	2 L	25–35 × 10^6^ cells/mL	Day 0–4: 1 VVD	Glucose prediction & control
			Day 4–17: 1.25 VVD	

A continuous cell bleed was implemented for cultivations C5 and C7 (see Table 1) to increase cultivation duration and achieve a more stable operation near steady-state. On-line permittivity measurements using a single-use impedance probe (BioPAT® Viamass, Sartorius, Germany) were correlated to the VCD based on empirical data specific for both cell line and medium (Grieb et al., 2019).

**FIGURE 1:**
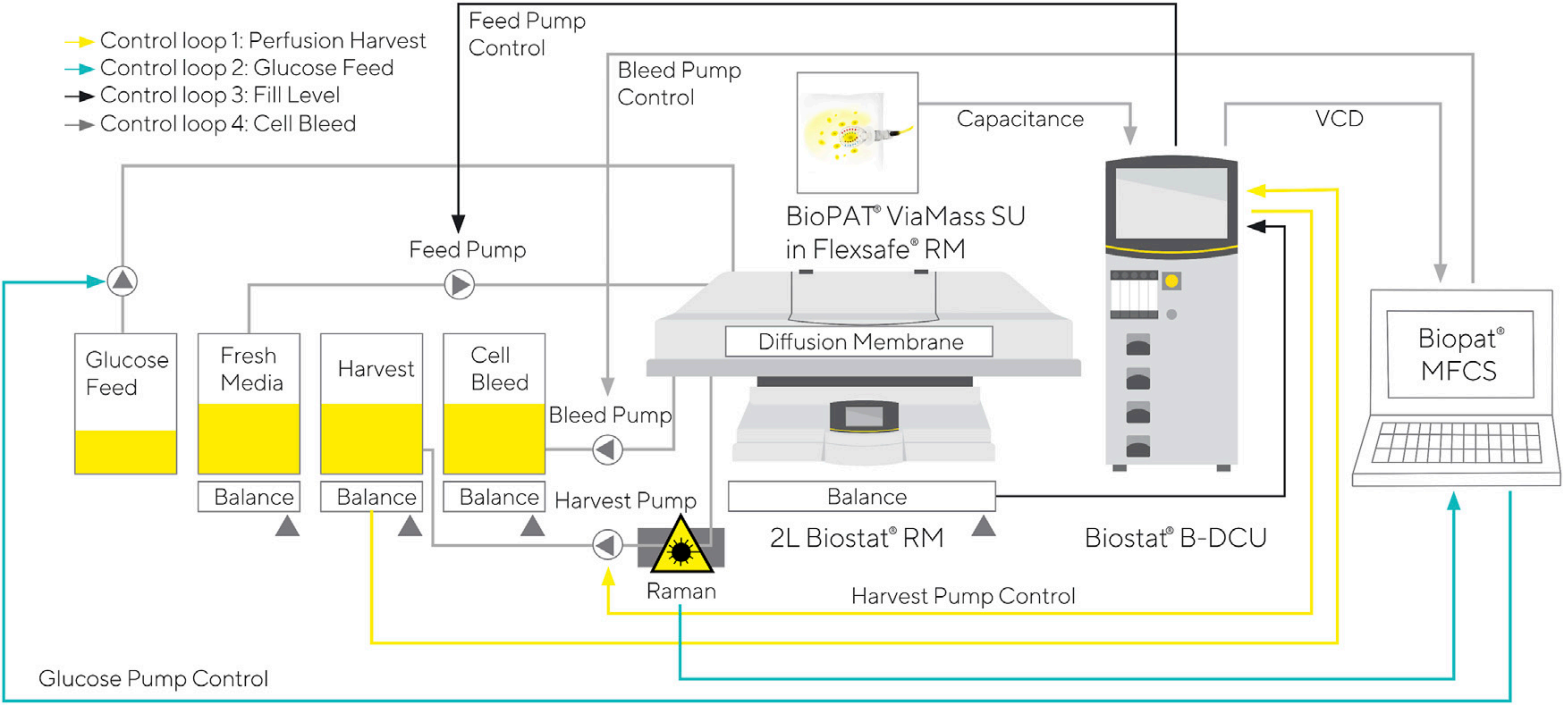
Control mechanism for RM perfusion cultivation with glucose control based in-line Raman measurements.

For the final perfusion cultivation C7 with in-line glucose control, perfusion medium without glucose was used as perfusion feed. Additionally, a perfusion medium with an increased glucose concentration (50 g/L) was used to control the glucose level. The detailed control mechanism is presented in Figure 1.

Off-line samples were taken manually from all cultivations. Cell growth (VCD, viability, and average diameter) were measured with a CedexHiRes Cell Counter (Roche, Germany). The pH, DO, glucose, and lactate levels were measured using a blood gas analyzer (ABL800 Basic, Radiometer, Germany).

### Raman Spectroscopy

For Raman measurements, an earlier prototype version of the BioPAT® Spectro Ambr measurement chamber (Sartorius, Germany) was incorporated in-line into the perfusion process’s harvest stream. Onto this chamber, a prototype probe-head was attached, which was connected to a HyperFluxPRO Raman spectrometer (785 nm, 495 mW, 200–3300 cm^−1^) (Tornado Spectral Systems, Canada) *via* optical fibers. In each run, the spectra were measured continuously with one data-point per minute (1000 ms × 60 avg; later averaged over five spectra), except for Run 6 (evaluation run), where each spectrum was directly measured over five minutes (1000 ms × 300 avg) to reach the same signal to noise ratio as the spectra used for model building.

### Chemometrics

Data from five different cultivations (C1–C5) were collected and used for building separate models for glucose, and lactate, respectively. These cultivations were intentionally run differently regarding the perfusion rates and profiles, as well as the bleeding regimen, to reduce possible correlations between glucose and batch maturity. A sixth run was used for model validation as an external test set before utilizing the model for actual glucose control.

For the first five model building runs, all one-minute spectra were first averaged over five minutes using a rolling mean. The averaged spectra were then imported into Easy Analytics (Sartorius, Germany) and merged with the off-line reference measurements. After reducing the overall dataset to the timepoints with reference values, it was exported to SIMCA 16 (Sartorius, Germany) for further evaluation and model building (see Supplementary Figure S1).

First, data pre-treatment was necessary to eliminate the fluorescence background and sensitivity differences that are hardware related such as probe mounting variations. Therefore, all spectra were baseline corrected with an asymmetric least squares algorithm (*p* = 0.05, λ = 10^7^) (Eilers and Boelens, 2005) before normalizing the spectra to the integral of the waterband around 1650 cm^−1^ (Lawaetz and Stedmon, 2009; Li et al., 2013). For each analyte of interest, i.e., glucose, and lactate, separate OPLS models (Orthogonal Projection to Latent Structures) were built. OPLS instead of classic PLS was chosen, as it increases the model interpretability while maintaining the same predictive power. This increase is achieved by removing variance in the X-Block (i.e. the spectra) which has no correlation to the variation in the Y-Block (i.e. the reference data)—or in mathematical terms removing systematic variation in X that is orthogonal to Y (Trygg and Wold, 2002; Eriksson et al., 2013).

In the case of glucose, prior to the model training, a second baseline correction (*p* = 0.05, λ = 10^7^) was applied just to the area of its strongest Raman signal (1093–1150 cm^−1^), as this has shown to improve the model robustness towards batch to batch variations significantly. For lactate, the spectra were reduced to areas that previous DoE studies have shown to be unique for the analyte. As spiking the samples with single analytes was not possible in this measurement set-up, this approach was chosen to reduce correlations between the two analytes of interest. For model evaluation, the focus lay on the cross-validation (cv) error, which gives a good first indication of the model’s predictive power. In this case, the dataset was split into five groups, each consisting of one batch, thus mimicking the model’s later use to predict future perfusion runs. For further information on the data-pretreatment, modeling procedure and especially the cross validation, please refer to Rowland-Jones et al. (2020).

Before using the model for actual glucose feed control, the model’s in-line monitoring capabilities were tested. Therefore, an additional run was conducted, and the models for glucose and lactate were applied to the raw spectra. In this instance, all the different pre-treatments (i.e., baseline correction and waterband normalization) used earlier were employed automatically by SIMCA to the new data as well.

Finally, the model was transferred to the spectrometer software (SpectralSoft, Tornado Spectral Systems, Canada) and loaded into the embedded SIMCA-Q (Sartorius, Germany) instance for in-line analyte prediction. The necessary data pre-treatments and prediction of the analyte concentration from the current spectrum was completed automatically without interaction from the user.

### Feed Control Automation

Utilizing the embedded SIMCA-Q instance in the spectrometer software, in combination with the previously build glucose model, the analyte concentration was automatically predicted for each measured spectrum. The communication between BioPAT^®^ MFCS 4.1 (Sartorius, Germany) and the Tornado Spectral Raman probe was established using the Python driver built into the BioPAT® MFCS 4.1 application as shown in Supplementary Figure S2. The implemented Python Controller can receive configurations, test the communication, and read process parameters and data, as required by the MFCS 4.1 Python driver. Further, MFCS 4 invokes this controller, especially the read functionality, in an adjusted sampling rate. This read functionality allows the read-in of process data over a middleware, using a RESTful application programming interface (REST-API). The middleware is caching the predicted values and is frequently communicating with the Tornado Spectral Software over ModbusTCP using the pymodbus package. The ModbusTCP interface offered by the Tornado Spectral Software provides specified registers. A register is used for storing generated univariate values.

The predicted glucose values are based on the OPLS model within SIMCA-Q. In the BioPAT® MFCS 4.1 application, these predicted glucose concentrations were then used as the input signal of a proportional-integral-derivative (PID) controller, which controlled the speed of the glucose feed pump depending on input and setpoint values (see Supplementary Figure S2). The control parameters were optimized during the process using an iterative approach before switching to a step response method as commonly described in the literature (Ziegler and Nichols, 1993; Chmiel et al., 2018).

## Results and Discussion

In this work, in-line Raman spectroscopy was applied to high cell density CHO perfusion processes. A flow-through cell was integrated into the perfusion harvest stream to evaluate the application of Raman spectroscopy for monitoring of various analytes. The in-line spectral data was correlated to the off-line reference data for glucose, and lactate, in separate OPLS models. In a final perfusion cultivation, the created glucose model was used for in-line process control.

### Analyte Model Building

Five different perfusion processes C1-C5 (see Table 1) were run to gather enough data points for building robust models for glucose and lactate. First, four N-1 perfusion cultivations C1–C4 were conducted. Their main goal is to achieve high VCDs to inoculate following N-stage production processes. Thus, no cell bleed is required. As this process was already established, it was used as a starting point for model building. Additionally, the wide ranges of glucose and lactate levels in those cultivations allow for improved model robustness compared to steady-state perfusions running at narrower concentration ranges.

**FIGURE 2:**
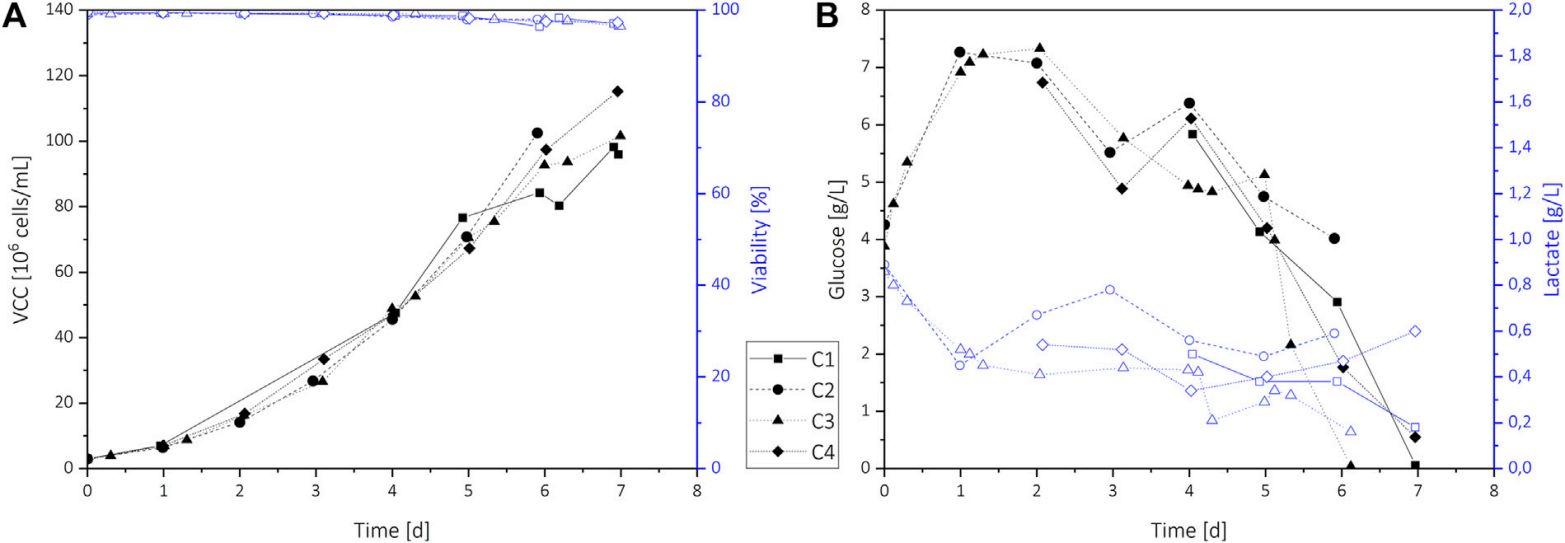
Process data of standard N-1 perfusion cultivations C1–C4 for analyte model building. The VCD and viability **(A)**, and the glucose and lactate concentration **(B)** for each cultivation are shown.

All perfusion runs C1–C4 showed very similar cultivation behavior achieving fast cell growth up to 100 × 10^6^ cells/ml and high viabilities above 95% (Figure 2A). Small deviations on days 6 and 7 resulted from differences in the processes, e.g., partial harvest (C1) or premature cultivation termination to inoculate following processes (C2). The use of 2 and 20 L perfusion bioreactor bags and an increasing perfusion rate (PR) from 1 VVD up to 5.5 VVD resulted in a wide range of harvest flow rates. Nevertheless, the same chamber and Raman probe could be utilized without any adverse effects on the collected spectra, demonstrating the suitability of this measurement approach for scale-independent transfer.

The glucose and lactate profiles are shown in Figure 2B. Initially, the remaining SMD medium was flushed out using a nutrient-rich perfusion medium at a constant PR of 1 VVD, causing an increase of the glucose concentration from 4 g/L to 7 g/L during the first two days. Increasing VCD and therefore increasing glucose consumption led to a glucose level of 5 g/L to 6 g/L on day 3. At this time, VCDs above 20 × 10^6^ cells/mL were achieved, which results in CSPR values of about 50 pL/(cell·d). As this value was identified as minimum CSPR in earlier perfusion trials (Janoschek et al., 2019), the PR was increased for all cultivations using varying profiles (see Table 1). To increase the glucose range within the cultivations for model building, the CSPR was reduced at the end of all cultivations. This resulted in glucose concentrations decreasing below 1 g/L on day 6 or 7, depending on process variations. For longer processes, an adaption of the PR to maintain a minimum CSPR of 50 pL/(cell·d) or an additional glucose feed would be necessary. Overall, the process shows very high and variable glucose concentrations ranging from 0 to nearly 8 g/L, which could not only affect product quality but also cell growth (Liu et al., 2015). As a fast growth rate up to very high cell densities is the main interest of seed train perfusion cultivations, these processes could also benefit from in-line glucose control.

During the initial batch phase, lactate accumulated in the bioreactor, which resulted in lactate levels of up to 0.9 g/L. With the perfusion start, lactate is partially washed out, and concentrations varied between 0.3 to 0.8 g/L. For cultivations C1 and C3 that showed deficient glucose levels below 0.5 g/L at the process end, lower lactate levels were observed, indicating a reduced lactate production or even consumption by the cells.

**FIGURE 3:**
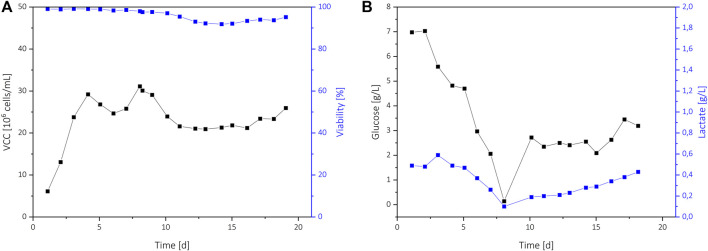
Process data of the N-stage perfusion C5 where a continuous cell bleed was implemented. The VCD and viability **(A)**, and the glucose and lactate concentrations **(B)** are shown.

An extended perfusion cultivation C5 with a continuous cell bleed was performed to transfer the process to a more N-stage focused cultivation where glucose-control would be beneficial (Figure 3). After reaching 30 × 10^6^ cells/mL on day 4, an on-line permittivity signal from a BioPAT® Viamass probe was used to control a bleed pump. This resulted in a steady VCD of 20 × 10^6^ cells/ml to 30 × 10^6^ cells/ml with high viability above 90%. Until day 8, a constant PR of 1 was applied, which resulted in a rapid decrease of the glucose level. On day 8, the PR was increased to ensure sufficient glucose addition, resulting in glucose levels up to 3.5 g/L.

**FIGURE 4:**
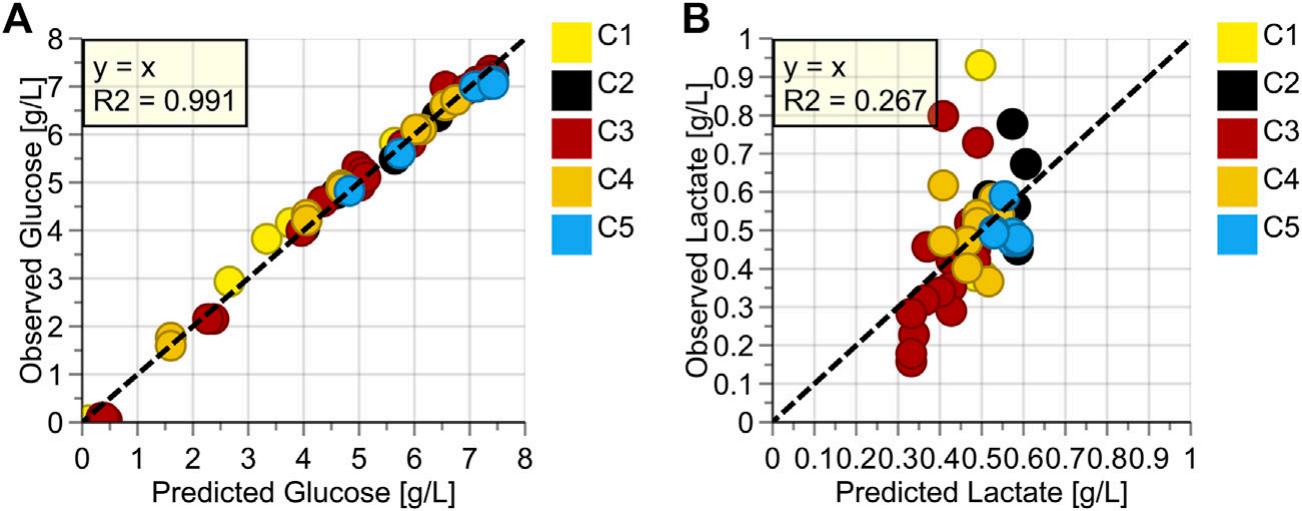
Observed vs. Predicted Plots for **(A)** Glucose (g/L), **(B)** Lactate (g/L); Colored according to Batch Number.

Quantitative OPLS-models for glucose and lactate were built (Figures 4A,B). For glucose, the reduced model to the dominant peak around 1125 cm^−1^ showed excellent performance with an R^2^ of 0.991 and a Root Mean Square Error of Cross Validation (RMSEcv) of around 0.2 g/L. For lactate, no valid model could be built, indicated by the low R^2^ value of just 0.267.

The good model performance of glucose agrees with previous findings with a similar spectrometer/probe/measurement chamber combination, which was integrated into an Ambr®15 system (Rowland-Jones et al., 2020). The in-line application of a Raman probe into the cell-free harvest stream of a perfusion process allowed for an excellent signal to noise ratio as no cells (i.e., scattering particles) are present in the sample. Furthermore, as soon as the perfusion medium exchanges the initial medium of the batch phase, its composition does not change significantly over time, resulting in a constant fluorescence background, which reduces baseline variation after baseline correction due to over or under correction. The different reactor sizes used and therefore resulting thirty-fold increase in harvest flow rates from 0.04 L/h to 1.25 L/h during the five cultivations, did not have any noticeable influence on the spectral quality. This should facilitate the model transfer to other scales that were not used in the model-building. As the flow-through measurement chamber is not implemented in the bioreactor but the perfusion harvest line, also a transfer to different perfusion bioreactors should be possible. In any case, the utilization of identical optics is required, which might require the chamber to be integrated in a by-pass at very high flow rates. Overall, the excellent model results indicate the suitability of the set-up for in-line glucose monitoring and, in conclusion also in-line glucose control.

For lactate, several reasons can be invoked as to why no valid model could be built (Figure 4B). First, the concentration range is relatively low. Experience gathered in previous trials has shown that at least ten times the model error (of a valid model) is needed for model building, so at least 2 g/L (Rowland-Jones et al., 2020). Second, most data-points are roughly between 0.4 g/L and 0.6 g/L. Ideally, the points should be spread out more equally over the entire concentration range so that each concentration is weighted equally in model building. Third, all higher concentration points originate from the beginning of the perfusion phase, when the medium is still mostly composed of the batch phase medium, which is significantly different from the perfusion medium. As the medium composition has shown to have a major impact on Raman spectra, this also negatively affects the model building (Santos et al., 2019). Overall, lactate is not a critical parameter in perfusion because of continuous media exchange and, therefore, no significant accumulation of this analyte. Consequently, continuous monitoring of the analyte is not necessary. If a valid model needs to be built, other actions can be taken, such as lactate spiking in off-line samples or including fed-batch cultivations with naturally occurring higher lactate concentrations.

### Model Validation

A sixth run C6 was conducted to test the glucose model and its predictive capabilities before using it for actual glucose control. As the data set did not fulfill basic requirements for generating an optimal lactate model, this analyte was not considered in the subsequent trials.

**FIGURE 5:**
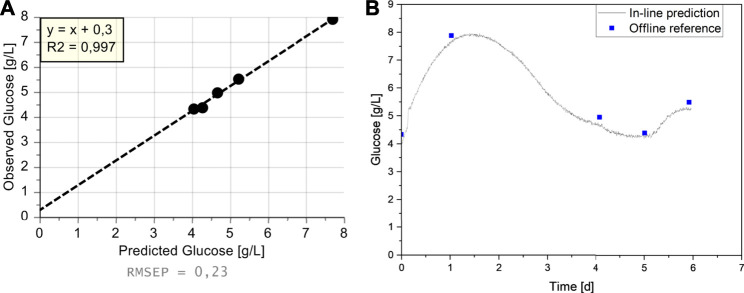
Predicted Glucose values in Cultivation C6; **(A)** Observed vs. Predicted Plot, **(B)** Predicted Glucose values from Raman Spectra (blue squares) and measured reference values (black line) over Batch Maturity.

With the Raman spectra generated during the sixth cultivation, the glucose value was predicted every five minutes, similar to the data acquisition rate during model building. The comparison with the off-line reference measurements reveals that the prediction was possible with a small Root Mean Square Error of Prediction (RMSEP) of 0.23 g/L and an R^2^ of 0.997 (Figure 5A). With this data, it is also possible to get a continuous trajectory of the glucose value for the entire cultivation in contrast to the sparse reference measurements (Figure 5B). As the perfusion process was performed mostly identical to the already described cultivations C1–C4, the glucose trajectory is similar (Figure 5B vs. Figure 2B). The perfusion is started after a batch phase in the seed medium, which led to a starting concentration of 4 g/L. A constant perfusion rate of 1 VVD was applied at the beginning of the process to flush out the seed medium. Combined with the low starting VCD of 2.5 × 10^6^ cells/ml, this resulted in an increase of the glucose concentration up to 8 g/L on day 2. As the VCD increased and therefore also the glucose consumption, the glucose level began to decrease on day 2. After reaching 20 × 10^6^ cells/ml on day 3, which equals a CSPR of 50 pL/(cell·d) at the applied 1 VVD, the perfusion rate was adjusted continuously to maintain a constant CSPR. This resulted in decreasing glucose concentrations until day 5, after which the bio-capacitance correlation factor was adjusted, therefore increasing the PR. As this increase was not part of the cultivations used for model building (C1–C5), it signifies a planned process deviation that can be utilized to test the model’s robustness and whether there is a possible correlation with the batch maturity. Since there is no evidence in the data indicating an influence of this variation, it confirms the previous findings that the Raman measurement is well suited for in-line monitoring of the glucose concentration, and in conclusion, also for in-line glucose control. The model error of around 0.2 g/L should be more than sufficient for the intended use, since a) it can be accounted for by sufficient safety margins or offset correction, and b) during regular operation, when a steady glucose concentration is reached, it also shows variations of around 0.2 g/L (compare to C5, day 10–15).

### In-Line Glucose Control Implementation

In the next step, the model was utilized for glucose control in an N-stage perfusion process with continuous cell bleed. The previously build glucose model was loaded into a SIMCA-Q instance, which was embedded in the Tornado measurement software. The in-line predicted glucose concentration was then transferred to BioPAT® MFCS, where it was employed as an input value for an MFCS PID Controller that controlled the speed of the glucose feed pump (Figure 1).

**FIGURE 6:**
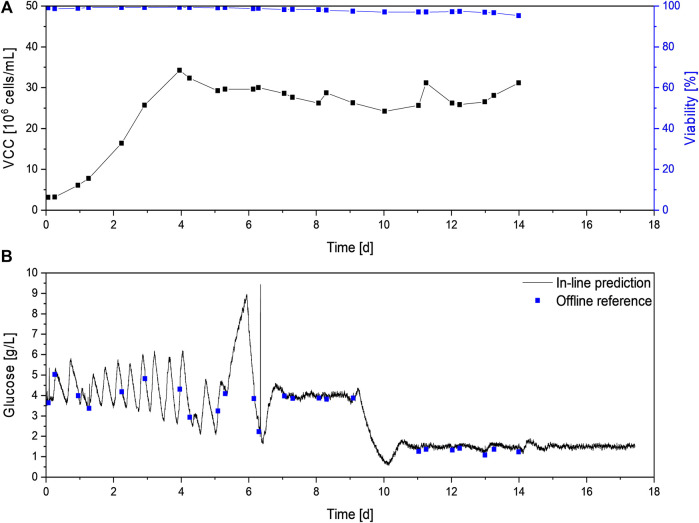
Process data of the N-stage perfusion C7 with in-line glucose control. The VCD and viability **(A)** as well as glucose in-line and off-line measurements **(B)** are shown. VCD, viability, and off-line glucose concentration were not measured after day 14.

After a batch phase in seed medium, perfusion was again started at 2.5 × 10^6^ cells/mL. Compared to the previously described cultivations C1-5, the starting glucose concentration was slightly lower at 3.6 g/L for C6 vs. 4 g/L in C1-C5 (Figure 6B vs. Figure 2B). Since initially a glucose-free perfusion medium was used for perfusion feed without any glucose addition, a dilution effect and consequently a lower starting glucose level was expected. Once the Raman flow-through probe was filled with medium and a steady permeate flow was achieved, glucose control was started with an initial set-point of 4 g/L. During the first 6 days of the cultivation, different settings for the newly implemented glucose controller had to be identified, resulting in substantial deviations from the set-point. Therefore, no robust glucose control could be achieved during this timeframe. However, model prediction closely followed the glucose concentration trend in a range of 2 g/L to 6 g/L. As no glucose limitation occurred, cell growth was not negatively affected, and a VCD above 30 × 10^6^ cells/ml could be achieved on day 4. Afterward, the continuous cell bleed based on on-line permittivity measurement was started, maintaining a VCD of 25 × 10^6^ cells/ml to 35 × 10^6^ cells/ml and high viability above 95% throughout the remaining cultivation (see Figure 6A).

Even with frequent changes in glucose levels, in-line glucose values could be obtained with high accuracy. The average deviation between both methods was 0.13 g/L, which is in accordance with the model error of 0.2 g/L previously described.

In order to improve control performance, on day five the technique to optimize control parameters was switched from an iterative approach to the step response method (Ziegler and Nichols, 1993; Chmiel et al., 2018). Therefore the controller was paused and a fixed control output, i.e. glucose feed pump rate, was applied, which led to drastic increasing glucose concentrations up to 9 g/L between day five and six (see Figure 6B). At this point the glucose feed was manually stopped to prevent negative effects on the cell culture, but theoretic control parameters could still be calculated by extrapolating the glucose concentrations. In addition, small changes were made to the set-up to improve the most challenging aspect of the control scheme—the long dwell time of analytes in the harvest line before reaching the sensor. As the Raman measurement was performed in the perfusion harvest stream and not in the bioreactor itself, the dwell time is directly dependent on the time required for the permeate to reach the Raman probe. Therefore, the dead time can be decreased by increasing the harvest flow rate and decreasing the harvest line diameter or the harvest line length from the bioreactor to the Raman probe. The harvest flow rate is directly dependent on the desired perfusion rate and, in conclusion, should not be changed. Therefore, the distance between the bioreactor and the Raman probe was shortened, reducing the process dead time by about ten minutes. This reduction combined with optimized control settings resulted in a stable control of glucose levels at 4 ± 0.4 g/L from day 7–9, after the controller was restarted between day 6 and 7. The constant glucose level was confirmed by the off-line reference measurements, which showed an average deviation of only 0.1 g/L to the predicted concentrations resulting in an overall RMSEP of around 0.1 g/L.

As previous studies showed improved product quality when maintaining glucose levels below 2 g/L (Berry et al., 2016), the control mechanism was challenged by further reducing the glucose set-point to 1.5 g/L on day 10. The new set-point was achieved after 24 h, and glucose levels were maintained at 1.5 ± 0.4 g/L for the remaining six days of the perfusion cultivation. Off-line measurements, which were performed until day 14 as reference, showed an average deviation between both methods of 0.2 g/L, which is in accordance with the determined RMSEP of 0.2 g/L. For both set-points, fluctuations in the glucose level larger than the model error were observed. This indicates that even tighter glucose control can be achieved by further optimization, e.g., by adjusting control parameters or glucose feed concentration.

In summary, Raman spectroscopy measurements in the perfusion harvest stream allow for accurate glucose monitoring at concentrations commonly occurring in biopharmaceutical processes with more than sufficient prediction errors around 0.2 g/L. No impact on harvest flow rate was observed. In combination with the possibility of implementing flow cells with similar optics in various tube diameters, scale-independent measurements could be achieved that, in consequence, might even be bioreactor independent. If the Raman probe is placed close to the reactor, process dead time can be reduced, enabling in-line control of glucose levels. It was shown that even low glucose concentrations of 1.5 g/L could be maintained over several days, providing a powerful tool for reduced glycation and improved product quality, as shown by Berry et al. (Berry et al., 2016).

## Summary and Outlook

In-line glucose control in a perfusion process was achieved by the combination of in-line Raman spectrometry, embedded chemometrics (SIMCA-Q), automated data transfer to the SCADA-software (MFCS 4.1), and implementation of a suitable controller that regulated a high-concentration glucose feed. With this set-up, it was possible to maintain the process continuously, first at 4 g/L, and later at 1.5 g/L, over several days with fluctuations of less than ±0.4 g/L. This enables the stable operation of the process at a given glucose set-point even if process deviations occur. Furthermore, the initial glucose model contained data from perfusion cultivations at 1–10 L working volume with perfusion rates ranging from 1 VVD to 5.5 VVD, resulting in an extensive range of perfusion harvest flow rates. As no effect of varying flow rates on measurement accuracy was detected, the measurement methods and glucose prediction model should be directly transferrable to large scale perfusion processes, as long as the underlying optical properties of the Raman probe stay similar. With the perfusion harvest line being independent of the used bioreactor, even the transfer to different reactor types, e.g., stirred tank bioreactors, should be possible and will be investigated in future works. For new perfusion processes, prediction models could be built in even smaller scales, e.g., Ambr® perfusion with a working volume of 250 ml and be transferred to control large scale processes. This would make the model building process much more time and cost-efficient, and a broader process range, i.e., in a DoE study, can be covered.

In future trials, the possibility of in-line monitoring of glucose and other analytes of interest, such as product titer, glutamine, or other amino acids, will be further investigated. The simultaneous measurement of metabolites and product titer is of special interest as it would allow for control of cultivation and purification parameters in integrated biomanufacturing processes.

Overall, this study demonstrates the suitability of Raman spectrometry as a PAT tool for in-line glucose control in prolonged perfusion processes, even if low and stable glucose concentrations are required. In combination with other on-line control tools, such as the BioPAT® Viamass integration for VCD control, an essential step towards fully automized continuous cultivation is done.

## Data Availability

The datasets presented in this article are not readily available because the raw data generated for this article are confidential. Data shown in the article are available upon request from the corresponding author. Requests to access the datasets should be directed to Johannes Lemke, johannes.lemke@sartorius.com.

## Supplementary Material

Supplementary Figure 1 Flow chart of OPLS model building from off-line reference and in-line Raman spectral data of perfusion cultivations C1-C5. Data sets were merged by capture time and baseline corrected prior to analyte-specific data pre-treatment.

Supplementary Figure 2 Flow chart of the implemented communication between the Raman Control System and the process control software BioPAT® MFCS. Predicted glucose values from generated Raman spectra were used to control the pump rate for glucose feed addition in perfusion cultivation C7.
