# Supplementary figures and images for: A Novel Approach for Non-Invasive Continuous In-Line Control of Perfusion Cell Cultivations by Raman Spectroscopy
Source: Front Bioeng Biotechnol. 2022 Apr 25;10:719614. doi: 10.3389/fbioe.2022.719614 (PMC9081366; doi:10.3389/fbioe.2022.719614)

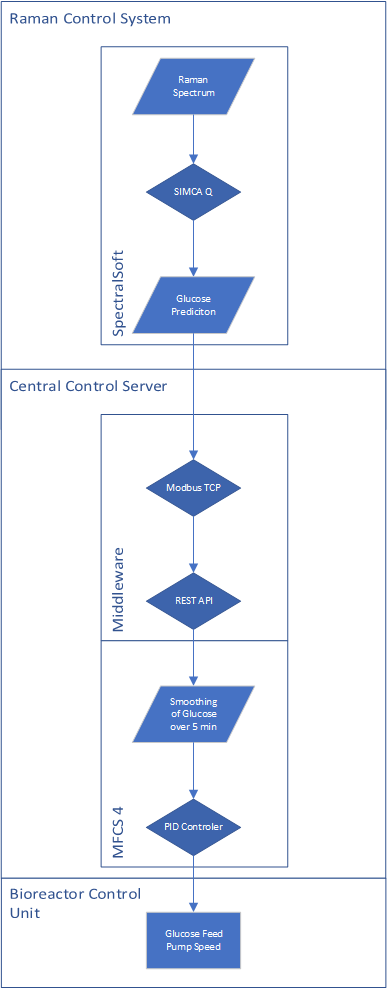

Supplement: Supplementary file 1 [file Image2.TIF]

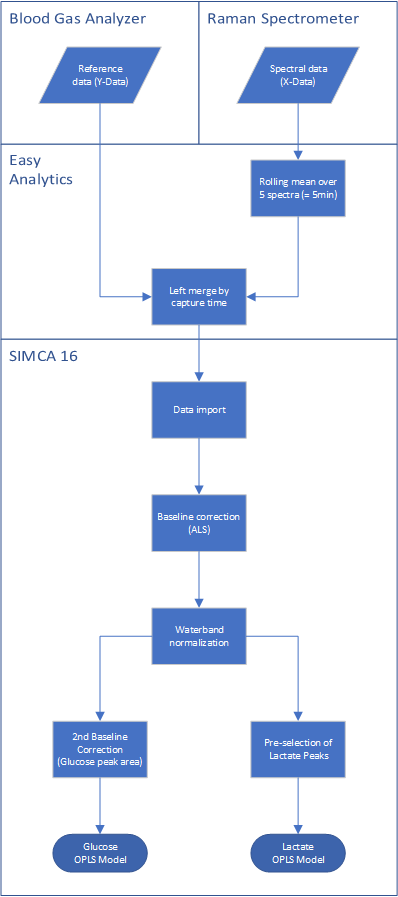

Supplement: Supplementary file 2 [file Image1.TIF]
